# Middle Cerebral Artery Stroke as Amusement Park Injury: Case Report and Review of the Literature

**DOI:** 10.3390/children4080064

**Published:** 2017-07-31

**Authors:** Abby Baumgartle, Laura Wolfe, Vinay Puri, Karen Moeller, Salvatore Bertolone, Ashok Raj

**Affiliations:** 1 School of Medicine, University of Louisville, Louisville, KY 40202 USA; albaum02@louisville.edu; 2 Department of Pediatrics, Division of Hematology/Oncology, 571 South Floyd St STE 445, Louisville, KY 40202, USA; laura.wolfe@louisville.edu (L.W.); sjbert01@exchange.louisville.edu (S.B.); 3 Department of Neurology, Division of Pediatric Neurology, University of Louisville School of Medicine, Louisville, KY 40202, USA; vinay.puri@louisville.edu; 4 Department of Radiology, Norton Children’s Hospital, Louisville, KY 40202, USA; karen.moeller@nortonhealthcare.org

**Keywords:** pediatric stroke, amusement park injury, middle cerebral artery stroke

## Abstract

Strokes as amusement park injuries are rare, but have been reported in the literature. Only about 20 cases of cerebrovascular accidents after amusement park visits have been described. We report a healthy 12-year-old boy who presented with facial droop, slurred speech, and inability to use his right arm after riding roller coasters at a local amusement park. He was evaluated and found to have a left middle cerebral artery (MCA) infarction. The patient was treated with anticoagulants and has recovered with no major residual symptoms. It is likely that his neurological symptoms occurred due to the high head accelerations experienced on the roller coasters, which are more detrimental to children due to immature cervical spine development and muscle strength. Early diagnosis of dissection and stroke results in a favorable prognosis. Providers and parents should be aware of the potential risk of roller coasters and act quickly on neurologic changes in children that have recently been to an amusement park.

## 1. Introduction

Strokes in children are a rare occurrence that can be due to diverse etiologies. Arterial ischemic stroke in children most commonly affects the middle cerebral artery (MCA) over other vessels [1]. Twenty cases of cerebrovascular accidents after amusement park visits have been described. These include reports of internal carotid artery (ICA) thrombosis, internal carotid artery dissection, vertebral artery dissection, and subarachnoid hemorrhage [2]. Amusement park rides encompass roller coasters, water slides, bumper cars, and spinning rides [3]. Cervicocephalic arterial dissection is a probable cause for stroke in children, especially those with family history of dissection, connective tissue disorders, and inherited defects in fibrillin and collagen [4,5]. We present a case of a 12-year-old boy who developed a left middle cerebral artery infarction as an amusement park injury and literature review of pediatric strokes in children without risk factors. Awareness of this risk is important so that both providers and parents can recognize signs and symptoms early to ensure best prognosis.

## 2. Patient Description

A previously healthy 12-year-old Caucasian male presented at his local emergency department complaining of sudden headache, right facial drooping, slurred speech, and dragging his right leg beginning the day before while he was chewing his breakfast. He was discharged home, with a diagnosis of a conversion disorder, but then re-presented the following day with the inability to use his right arm along with his prior symptoms. The patient’s family denied any injury or trauma, however, the patient was at a local amusement park the day before his symptoms began. They reported spending 9 h at the park riding roller coasters. He may have had symptoms beginning at the park because he was found struggling in the waves of the park’s wave pool. At his second return to the emergency department (ED), a computed tomography (CT) of the brain was performed, which was abnormal. A brain magnetic resonance imaging (MRI) was done for further evaluation. Diffusion weighted images and apparent diffusion coefficient images were obtained. His imaging showed concern for a left middle cerebral artery stroke. The patient was transferred to our facility for further care.

A neurologic exam showed the patient to be distractible with difficulty maintaining his attention. His speech was slurred. Extraocular movements were intact with equal and reactive pupils bilaterally. All cranial nerves were normal except a definite right facial droop. His strength was 4/5 in right sided arm abduction, flexion, extension, hand pronation, grip, knee flexion, and knee extension. Strength was 5/5 for the same exam on the left side. He exhibited flaccid muscle tone on the right side. He was hyper-reflexive at 3+ for his right triceps, brachioradialis, patellar, and ankle reflexes while being 2+ for all reflexes on the left side. Light touch was intact in all dermatomes examined. He has no known history of coagulation disorder or thrombotic disease.

**Figure 1:**
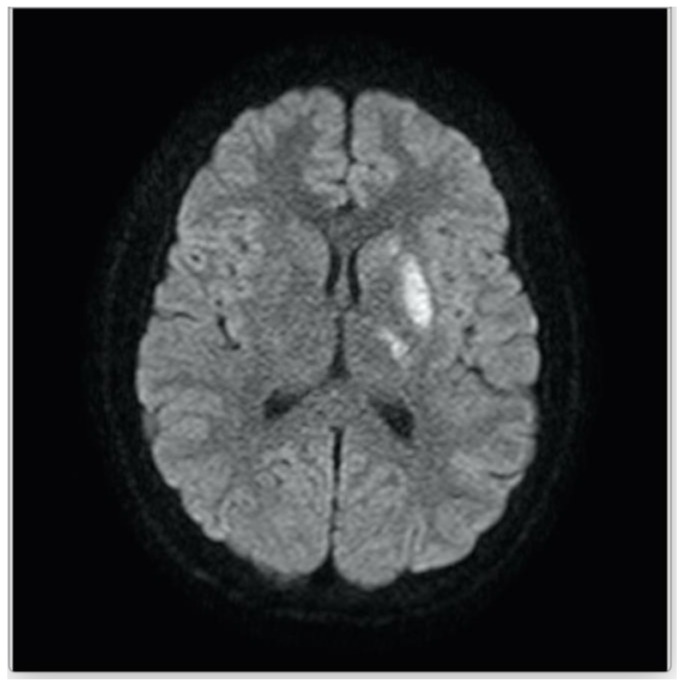
Diffusion weighted image shows restricted diffusion in the left lentiform nucleus, ventrolateral thalamus and caudate head, consistent with acute ischemia or infarct in the distribution of the left middle cerebral artery (MCA).

**Figure 2:**
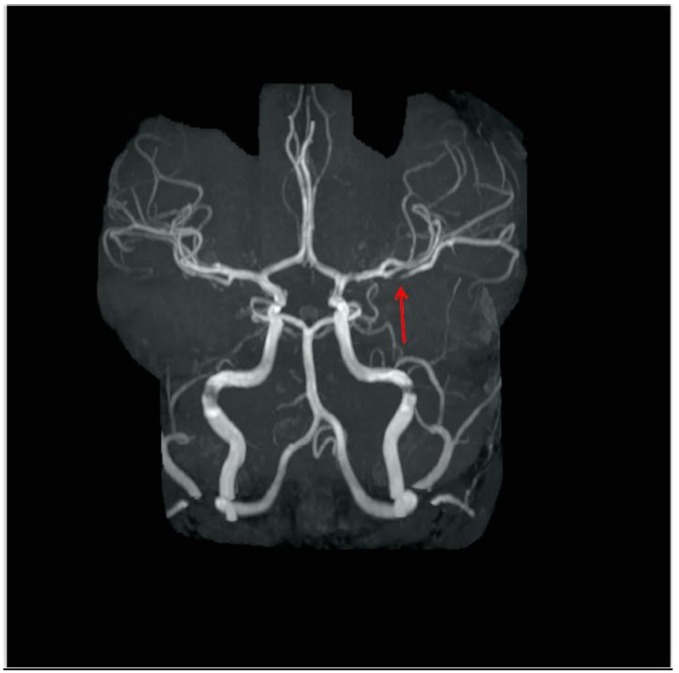
Magnetic resonance angiogram (MRA) shows a defect in the posterior branch of the left MCA four days after roller coaster ride.

The patient underwent laboratory testing including a complete blood count, which was unremarkable with a white blood cell count of 6.30 × 10^3^/μL, hemoglobin of 13.3 g/dL, hematocrit of 37.1% and platelet count of 217 × 10^3^/μL. At our facility, a repeat MRI/magnetic resonance angiogram (MRA) of the brain showed small areas of acute infarction in distribution of the left middle cerebral artery that was stable compared to the prior MRI done the day before in his local ED (Figure 1). There was also a focal abnormality of the proximal left M2 branch suggestive of thromboembolic disease (Figure 2). An electroencephalography (EEG) performed was suggestive of left frontal cerebral dysfunction. An MRA of the neck indicated normal flow in the common carotids and internal carotids without evidence of dissection in these vessels. An echocardiogram with bubble study indicated no structural or functional abnormality.

A full hypercoagulability analysis was completed including lupus anticoagulant, factor V Leiden, protein C activity, protein S antigen, partial thromboplastin time (PTT), prothrombin time (PT), international normalized ratio (INR), fibrinogen, d-dimer, Antithrombin III, homocysteine, cardiolipin IgG and lipoprotein A. All labs were unremarkable, except protein C level was low at 71% normal. He was also found to be heterozygous for methylene tetrahydrofolate reductase (MTHFR) mutation, but with normal fasting homocysteine levels this was determined to not be the likely etiology of his hypercoagulable state.

**Figure 3:**
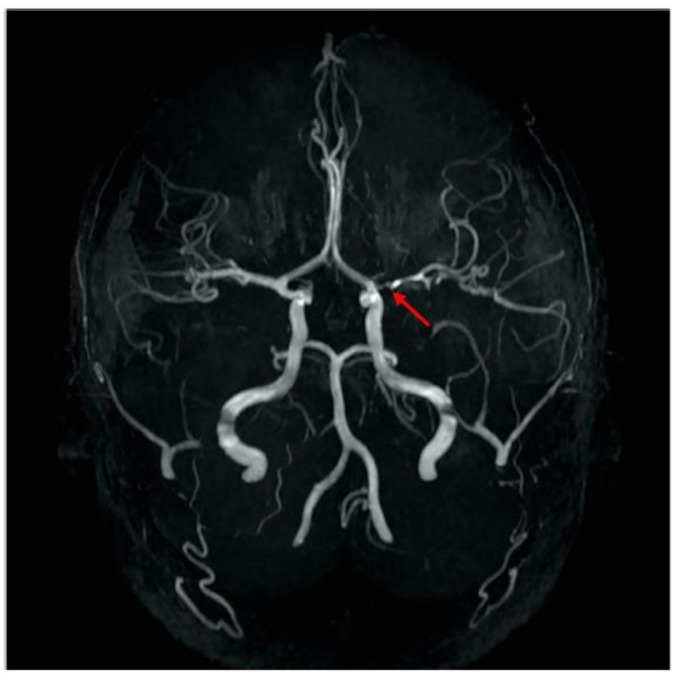
MRA follow up shows narrowing of the left M1 artery four months after roller coaster ride.

The patient was diagnosed with a left middle cerebral artery stroke from a blood clot of the posterior inferior branch of the left MCA. Initial anticoagulation therapy included subcutaneous enoxaparin which was transitioned to aspirin daily. He was discharged to a local rehabilitation facility where he stayed as an inpatient for three weeks, undergoing integrated and intensive therapy. His neurologic exam post hospital discharge showed mild asymmetry in the right facial muscles, mild weakness on right side, arms greater than legs, mild pronator drift, and hyper-reflexia on the right side. These symptoms and signs have nearly resolved. A follow up MRA four months after his roller coaster ride showed narrowing of the left M1 artery (Figure 3).

## 3. Discussion

Approximately 92,885 children under the age of 18 years old were treated in the emergency department for amusement park related injuries in the United States between 1990 and 2010 [3]. However, Loder et al. [6] state that only 7% of these injuries are serious enough to require overnight treatment. The majority are minor injuries such as fractures, sprains/strains and dislocations [6]. As technology improves, roller coasters are being designed to create a greater thrill by incorporating higher vertical drops and thus greater G forces [7]. Traditionally, roller coasters achieved peak car accelerations around 2–3 G, but manufactures have created rides that deliver more than 6.5 G [8]. These G forces cause the head to accelerate and rotate during the ride. This rapid head rotational acceleration can cause significant brain injury, such as diffuse white matter axonal injury. At high accelerations, even tissue tears and vascular disruption can occur [9,10]. These G-forces are not sustained and only apply accelerations in different direction for less than three seconds. If an individual sustains G forces of around 5–9 G for an extended period of time, greater than 40 s, unconsciousness will occur due to decreased blood flow to the brain [7].

Strokes in children are somewhat rare, but arterial dissection can be a cause of stroke in the pediatric age groups [11]. Cervicocephalic arterial dissections account for 2% of ischemic strokes for the general population and 5% of pediatric strokes [12]. Roller coasters have been linked with dissections of the internal carotid artery, vertebral arteries and middle cerebral artery resulting in strokes [13]. The vertebral arteries can be dissected due to rotational forces at the third segment of the artery at the C1–C2 vertebral levels [14]. The internal carotid artery has been shown to reach its maximal stress at 90 degrees of lateral rotation or 45 degrees of hyperextension [5]. Hyperextension, rotation, or lateral flexion of the neck can stretch and compress the ICA and vertebral arteries against the bony portions of the cervical spine, such as the transverse processes, vertebral bodies or styloid process. All of which can cause dissection of the artery [2,5]. Dissections cause stenosis of the true lumen and disturbance of the epithelium slowing the flow of blood and creating turbulence. As a result, a local thrombus can form leading to embolism and stroke [14].

Case reports have shown numerous incidences of strokes in pediatric and adult patients after amusement park rides (Table 1). The cohort of pediatric cases reviewed included a four-year-old who experienced a right internal carotid artery dissection causing right MCA territory infarction and an 11-year-old left vertebral artery dissection. Both had been on roller coasters prior to the onset of their neurological symptoms [5,15]. Children are more vulnerable to injury on a roller coaster due to anatomical development of the pediatric cervical spine. The cervical spine will not be as developed as adults until 8–10 years of age [5]. Due to ligament laxity, shallow and angled facet joints, and less developed vertebral bodies in children, hypermobility of the neck is possible. More instability is created by the larger pediatric head with weaker neck muscles. The fulcrum of motion of the pediatric cervical spine is at the C2–C3 level and at C5–C6 in adults [5]. All of these are contributing factors that may put children at more risk for injury with sudden neck movements.

The diagnosis of dissection in children is often delayed due to the vague symptoms of headache, neck pain and dizziness [11]. Dissection should always be included on the differential when a patient either young or old presents with ischemic symptoms without the typical risk factors for stroke [4]. Stroke specifically after a roller coaster ride should be worked up as possible vessel dissection until ruled out [14]. Prognosis of a child presenting with stroke is related to the severity of the presenting infarction [4]. Patients recovering from strokes after dissection are typically started on anticoagulants in an effort to prevent a recurrence. If the diagnosis of dissection can be made prior to an infarction, the outcome is more promising [15]. Specifically recognizing dissection of an internal carotid artery is time sensitive because one-third of patients are at risk of developing a hemispheric stroke within the first week [11].

Roller coasters at most parks have posted warnings for individuals with high blood pressure, back or neck conditions, recent surgery, pregnancy, motion sickness/dizziness and heart conditions, but most do not warn of possible neurologic complications after the ride [17]. Therefore, parents do not recognize symptoms that they can correlate to an amusement park ride. Delay in treatment can occur if parents inadvertently postpone medical treatment. Recognition of common signs of stroke in both children and adults—such as facial droop, arm weakness, and speech disturbance—is essential in ensuring the best outcomes [18]. Providers should be aware of these risks and be suspicious of headache, neck pain and neurologic symptoms in patients with a recent history of sudden neck movements such as during roller coaster rides [16].

## 4. Conclusions

Roller coaster rides have been linked to pediatric strokes, as was seen in our case and several more in the literature. Providers should be aware of this possibility in order to diagnose early for the best prognosis. Parents should be informed so that they can recognize symptoms like facial droop, arm weakness and speech disturbance that will need medical treatment.

## Figures and Tables

**Table 1:** Cases of infarctions and dissections in adults and children after amusement park rides.

Case	Year	Sex/Age	Clinical Symptoms	Diagnosis
Burneo [14]	2000	M/30 years old	Diplopia and cervical pain	Left occipital infarction; left vertebral artery dissection
Lascelles [15]	2001	M/11 years old	Sudden headache, hemiparesis, ataxia, dizziness, nausea and vomiting	Left cerebellar infarct and infarction of both thalami; left vertebral artery dissection
Schneck [12]	2008	F/34 years old	Neck pain, vertigo, blurred vision, and middle horizontal diplopia	Bilateral cervical vertebral dissections
Arat [11]	2011	F/35 years old	Right sided neck pain and frontal headache	Bilateral ICA and vertebral artery dissections
Leitao [16]	2012	M/22 years old	Occipital headache, vertigo, nausea, vomiting and ataxia	Infarct of the right cerebellum; dissection of the right vertebral artery at C1–C2
Kurita [2]	2014	M/39 years old	Headache, sudden right hemiplegia and aphasia	Infarction of left MCA territory; ICA and MCA dissection
Nouh [5]	2015	M/4 years old	Left facial droop, vomiting, inability to walk and left sided weakness	Right MCA infarction; right internal carotid artery dissection
Baumgartle *	2016	M/12 years old	Headache, facial drooping, slurred speech and right sided weakness	Left MCA infarction

M: Male; F: Female; ICA: Internal Carotid Artery; MCA: Middle Cerebral Artery. * Current case report.
